# Numerical Study to Enhance the Sensitivity of a Surface Plasmon Resonance Sensor with BlueP/WS_2_-Covered Al_2_O_3_-Nickel Nanofilms

**DOI:** 10.3390/nano12132205

**Published:** 2022-06-27

**Authors:** Shivangani, Maged F. Alotaibi, Yas Al-Hadeethi, Pooja Lohia, Sachin Singh, D. K. Dwivedi, Ahmad Umar, Hamdah M. Alzayed, Hassan Algadi, Sotirios Baskoutas

**Affiliations:** 1 Department of Electronics and Communication Engineering, Madan Mohan Malaviya University of Technology, Gorakhpur 273010, India; shivangani9673@gmail.com; 2 Department of Physics, Faculty of Science, King Abdulaziz University, Jeddah 21589, Saudi Arabia; malhabrdi@kau.edu.sa (M.F.A.); yalhadeethi@kau.edu.sa (Y.A.-H.); hmalzayed@stu.kau.edu.sa (H.M.A.); 3 Photonics and Photovoltaic Research Lab, Department of Physics and Material Science, Madan Mohan Malaviya University of Technology, Gorakhpur 273010, India; sachin111iitp@gmail.com; 4 Department of Chemistry, College of Science and Arts, Najran University, Najran 11001, Saudi Arabia; 5 Promising Centre for Sensors and Electronic Devices (PCSED), Najran 11001, Saudi Arabia; hassan.algadi@gmail.com; 6 Department of Electrical Engineering, College of Engineering, Najran University, Najran 11001, Saudi Arabia; 7 Department of Materials Science, University of Patras, 265 04 Patras, Greece; bask@upatras.gr

**Keywords:** surface plasmon resonance sensor, blue-phosphorus tungsten di-sulfide, Al_2_O, nickel, sensitivity

## Abstract

In the traditional surface plasmon resonance sensor, the sensitivity is calculated by the usage of angular interrogation. The proposed surface plasmon resonance (SPR) sensor uses a diamagnetic material (Al_2_O_3_), nickel (Ni), and two-dimensional (2D) BlueP/WS_2_ (blue phosphorous-tungsten di-sulfide). The Al_2_O_3_ sheet is sandwiched between silver (Ag) and nickel (Ni) films in the Kretschmann configuration. A mathematical simulation is performed to improve the sensitivity of an SPR sensor in the visible region at a frequency of 633 nm. The simulation results show that an upgraded sensitivity of 332°/RIU is achieved for the metallic arrangement consisting of 17 nm of Al_2_O_3_ and 4 nm of Ni in thickness for analyte refractive indices ranging from 1.330 to 1.335. The thickness variation of the layers plays a curial role in enhancing the performance of the SPR sensor. The thickness variation of the proposed configuration containing 20 nm of Al_2_O_3_ and 1 nm of Ni with a monolayer of 2D material BlueP/WS_2_ enhances the sensitivity to as high as 374°/RIU. Furthermore, it is found that the sensitivity can be altered and managed by means of altering the film portions of Ni and Al_2_O_3_

## 1. Introduction

A method named surface plasmon resonance has arisen as an incredibly sensitive procedure for recognizing a very significant alteration in the refractive indexes of a detecting medium while communicating with the metal layer [1,2,3]. Enzyme detection, drug detection, medical diagnostics, and food safety are some of the biosensing applications of SPR-based biosensors [4,5,6,7,8]. Without any need for biomolecule labeling, a minute change in the refractive index (RI) can be detected in the detecting medium [9]. SPR is a highly sensitive technology that can detect very small fluctuations in the refractive index (RI) for biomolecule absorption of the order of 10^−7^ on the sensing interface. At the metals’ dielectric contact, the collective oscillation of free electrons generates a transverse magnetically polarised electromagnetic wave known as a surface plasma wave (SPW). SPWs are made from metals with negative permittivity, such as gold, silver, copper, and aluminium, as well as dielectric materials, which can be liquid, gas, or solid. Researchers suggest that the Kretschmann arrangement brings about the productive coupling of light first from the crystal to the metal region, which is rooted in attenuated total reflection (ATR) [10]. The metallic regions of surface plasmons must be energized by the p-polarized light, while the s-polarised part is utilized as a source for the reference signal [11].

The SPR is acquired by the horizontal part of the evanescent wave (k_ev_) being stage-matched to the surface plasmon wave vector (k_sp_). For example,:

$$k_{\mathrm{ev}}=\sqrt[{k0}]{\in p}k_{p}\sin\theta_{\mathrm{res}}{}_{}=k_{\mathrm{sp}}$$

where the incident wave vector is addressed by k_0_ = ω/c, crystal permittivity is addressed by ∈p, and the resonance point–angle is addressed by θ_res_. Because of the total change of the p-polarized wave to the SP waves, the SPR condition causes a reduction in the depth of mirrored light (R).

In SPR sensors, normal metal layers of gold, silver, copper, and platinum are utilized. These are also plasmon-active metals that are used to generate surface plasmon waves in the sensing medium. The most promising metal is gold (Au), which has excellent optical properties, good chemical stability, and high oxidation and corrosion-resistant properties, although gold is the most expensive metal and lowers the biological molecules’ absorption rate as compared to Ag metal [12,13,14]. The precision of an SPR sensor built from Ag film is better than that of an Au-film-based sensor. Because silver film is less costly than gold film, and as it was observed that silver has better sensitivity than Au metal, silver’s SPR curve dip is narrower than gold’s, meaning the sensitivity is improved. However, the chemical solidity problem of Ag should be alleviated, and the protecting layers must be explored for favorable optical properties [15,16,17]. As a result, this work introduces a novel aluminium oxide dielectric material to improve the SPR performance (Al_2_O_3_). It must be used to improve the SPR biosensor’s performance parameters, such as the figure of merit (FOM), sensitivity, and so on. In fact, Al_2_O_3_ is widely used in mechanical areas due to its well-known properties, such as its superior corrosion resistance, high ductility, and high hardness, as well as in optical devices due to its high transparency and low refractive index. Certain 2D materials, such as graphene, WS_2_, and the 2D heterostructure BlueP-WS_2_, are the implicit aspirants used as a self-productive layer to give stability and boost the SPR sensor as it is slowly oxidized by air at room temperature and are considered corrosion-resistant. Transition metal dichalcogenide (TMDC) substances have a curial position in SPR sensors. PtSe_2_, Ti_3_C_2_Tx-Mxene, blue phosphorus, black phosphorus, and transition metal dichalcogenides (TMDC) are examples of substances that have stood out in the past twenty years due to their notable optical and electrical nature and have been used to make optoelectronic devices. Forty unique synthetic substances are presently incorporated in the TMDC family. MX_2_ is another identifier for them, where M denotes metals such as tungsten, molybdenum, and niobium; and X denotes the chalcogen substances such as sulfur, selenium, and tellurium. MX_2_’s monolayer has three nuclear layers, with an alternate metal layer embedded between two chalcogen substance layers [18]. These nanomaterials collaborate with the metal layer and enhance the co-operation of the particles. If the oxidation of Al_2_O_3_ (2D material) can be minimized by means of coating it with another layer, we can use it as a high-sensitivity sensor. Because of its refractive index, Al_2_O_3_ ensures that the biosensor’s performance is no longer affected. The homogeneity of the Al_2_O_3_ layer is a characteristic that leads to sensitivity enhancements [19]. Nickel (Ni), a ferromagnetic metal, is also gaining interest due to its notable magneto-optical and magnetic properties and being a good light absorber. Using inert magnetic metals minimizes the cost of the SPR sensor while simultaneously considerably improving its performance [20,21,22].

Moreover, because the TMDCs and blue phosphorene have a similar hexagonal crystal-like structure, BlueP/TMDCs can be formed [23]. Therefore BlueP/WS_2_‘s heterostructure shows greater sensitivity. The sensing medium, also known as a sensing analyte, is the outermost layer in the proposed SPR biosensor design. There are 2 main SPR geometry configurations: the Kretschmann configuration and Otto’s design. The Kretschmann configuration’s benefits over Otto’s arrangement have made it more widely applicable [24]. The SPR-based sensor’s biosensing application covers the foundations of detecting the concentrations of biological things on a very small scale, such as bacteria, viruses, DNA, and proteins. Apart from biosensing and biomolecular analysis, the SPR sensor may also be used to detect nanostructured film depositions, as well as to quantify displacement and angular position [25,26,27].

In the present paper, calcium fluoride glass crystal (CaF_2_) is taken for the proposed SPR biosensor since it gives maximal sensitivity and an enormous change in reflectivity when contrasted with different crystals (BK7, SF10, SF11, and so forth). A framework for an SPR sensor based on heterostructure containing Al_2_O_3_, BlueP/WS_2_ with metal (Ag), and Ni (Nickle) is proposed in the present work to achieve improved sensitivity by altering the thickness of the Ni layers. The results demonstrate that adding the Al_2_O_3_ layer and BlueP/WS_2_ to this structure boosts the sensitivity substantially. In this work, COMSOL Multiphysics 5.3a and MATLAB 2016a softwares are used to draw the plot of the reflectance curve, sensitivity curve, and electric field curve, which are calculated using these software programs.

**Figure 1:**
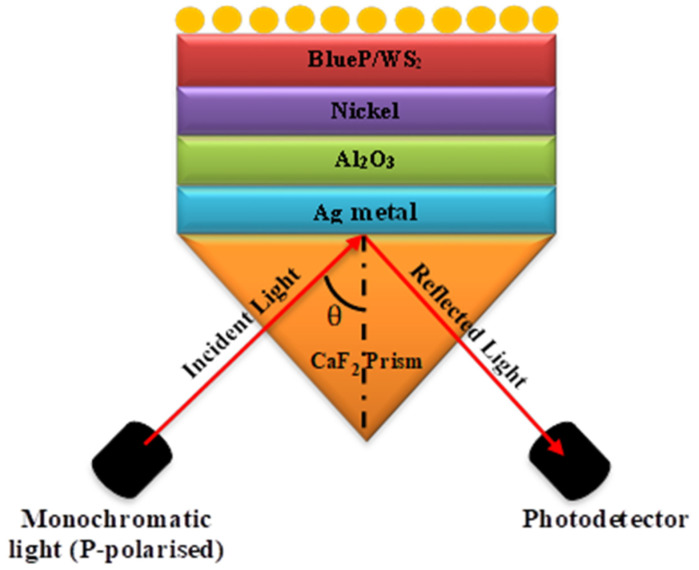
Sketch diagram of the proposed SPR biosensors.

## 2. Mathematical Modeling for the Proposed SPR Biosensor

### 2.1. Device Structure

In the present paper, the SPR sensor is composed of multiple layers primarily based on the Kretschmann configuration, in which the prism of CaF_2_, Ag as a metal, Al_2_O_3_ (diamagnetic material), BlueP/WS_2_, and SM on the top layer is used as shown in Figure 1. Table 1 refers to the width and different refractive indexes of the layers.

The structural diagram using Ag-Al_2_O_3_-Ni-BlueP/WS_2_ is depicted in Figure 1 for this model. The 633 nm wavelength is utilized in SPR sensors for optimal results. The CaF_2_ prism is employed in metal Ag with a refractive index of 1.4329 and a thickness of 50 nm, in Al_2_O_3_ with a thickness of 20 nm, in nickel with a thickness of 1 nm, and in BlueP/WS_2_ with a thickness of 0.75 nm. Finally, the sensing medium has a refractive index of 1.330–1.335. The variation in the detecting medium is caused by adsorption, which occurs when biomolecules in the sensing medium contact with the BlueP/WS_2_ layer, causing the sensing medium’s RI to alter. The first film is the CaF_2_ prism, whose refractive index value may be computed using the Sellmeier relation.

(1) $$n^{2}=1.33973+\frac{0.69913\times \lambda^{2}}{\lambda^{2}-{\left(0.09374\right)}^{2}}+\frac{0.11994\times \lambda^{2}}{\lambda^{2}-{\left(21.18\right)}^{2}}+\frac{4.35181\times \lambda^{2}}{\lambda^{2}-{\left(38.46\right)}^{2}}$$

Here, ‘λ’ is the wavelength in nanometers. The RI of Ag is calculated using the Drude–Lorentz model:

(2) nmetalλ=1−λ2×λCλP2λC−λ×i12                                 

where λ_c_ and λ_P_ are the collision and plasma wavelengths, respectively. These are dispersion coefficients, while the used wavelength is 633 nm.

For Ag, λ_P_ = 145.41 nm and λ_C_ = 176.14 nm [33]. The transfer matrix method for the n-layer modeling and Fresnel equations is used throughout the numerical analysis. Using the reflectance curve, all the SPR sensor’s performance characteristics in MATLAB software are evaluated. The graph is drawn using Origin software for all parameters and the FWHM values. Here, additionally compared distinct SPR sensors with the proposed model are mentioned in Table 2.

### 2.2. Mathematical Expression for Reflectivity

The transfer matrix approach is utilized in the present work to obtain the reflection coefficient of the projected multilayer design (crystal, metal, Al_2_O_3,_ Ni, and BlueP/WS_2_ film). For the calculation of the reflectivity of the reflected light, the matrix approach for the N-film design is used. This method is quick and easy to use, and it does not involve any approximation. Along the z-axis, the layer thicknesses, dk, is considered. The k^th^ layer’s dielectric constant and RI are denoted by k and n, respectively. The tangential fields at Z = Z_1_ = 0 are expressed in terms of the tangential field at Z = Z_N−1_ using the boundary condition [34]:

(3) $$\left[\begin{array}{c} U_{1}\\ V_{1} \end{array}\right]=M\left[\begin{array}{c} U_{N-1}\\ V_{N-1} \end{array}\right]$$

The places U_1_ and V_1_ address the tangential element of electric and magnetic fields, respectively, and the first and last layer of boundary are denoted by U_N−1_,V_N−1_, respectively. M_ij_ is the element for which the characteristics matrix is as follows [35]:

(4) $$M_{\mathrm{ij}}={\left(\prod_{k=2}^{N-1}M_{k}\right)}_{\mathrm{ij}}=\left[\begin{array}{cc} M_{11} & M_{12}\\ M_{21} & M_{22} \end{array}\right]$$

(5) $$M_{k}=\left[\begin{array}{cc} \cos\beta \kappa & \left(-𝒾\sin\beta \kappa \right)/q_{k}\\ -iq_{k}\sin\beta \kappa & \cos\beta \kappa \end{array}\right]$$

(6) qk=ukεk1∕2cosθκ=εk−sinθ1n1212εk

and

(7) qk=ukεk1∕2cosθκ=εk−sinθ1n1212εk

Following the mathematical steps, one can attain the reflection coefficient for p-polarized light, which is given below:

(8) $$r_{p}=\frac{\left(M_{11}+M_{12}q_{n}\right)q_{1}-\left(M_{21}+M_{22}q_{n}\right)}{\left(M_{11}+M_{12}q_{n}\right)q_{1}+\left(M_{21}+M_{22}q_{n}\right)}$$

The multilayer configuration of the reflectivity R_p_ is given as:

(9) $$R_{p}={\left|r_{P}\right|}^{2}$$

**Figure 2:**
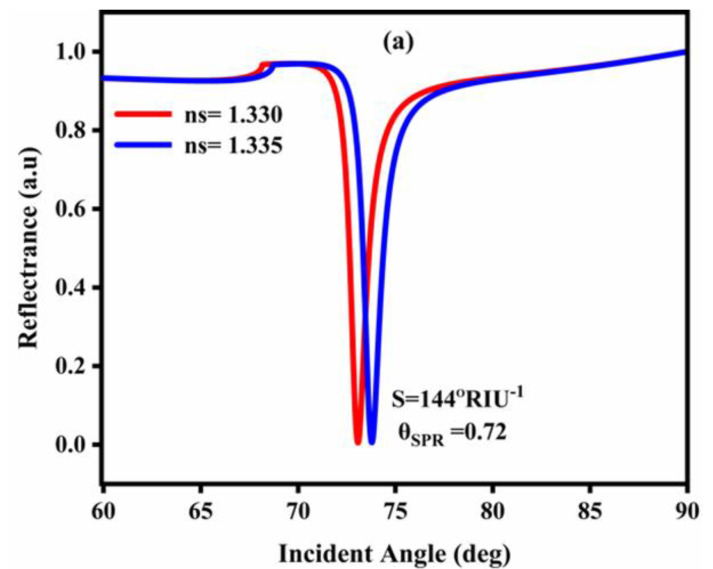

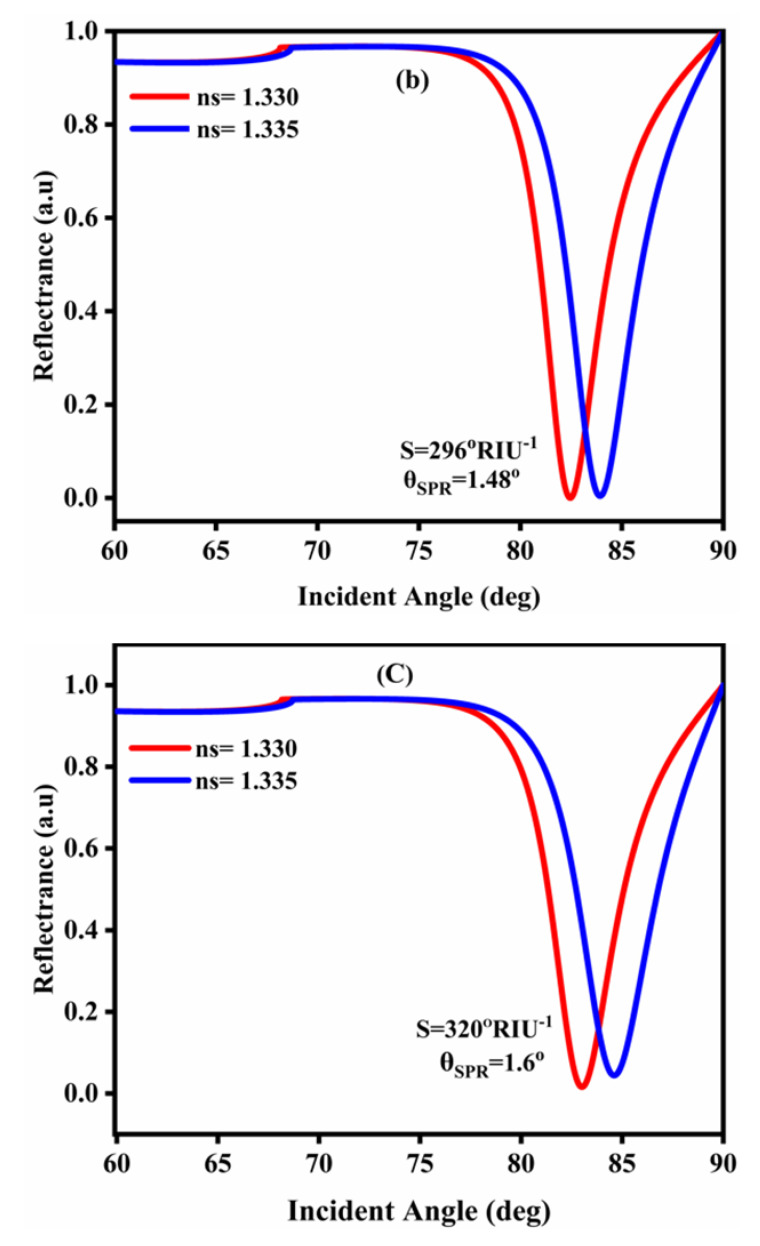

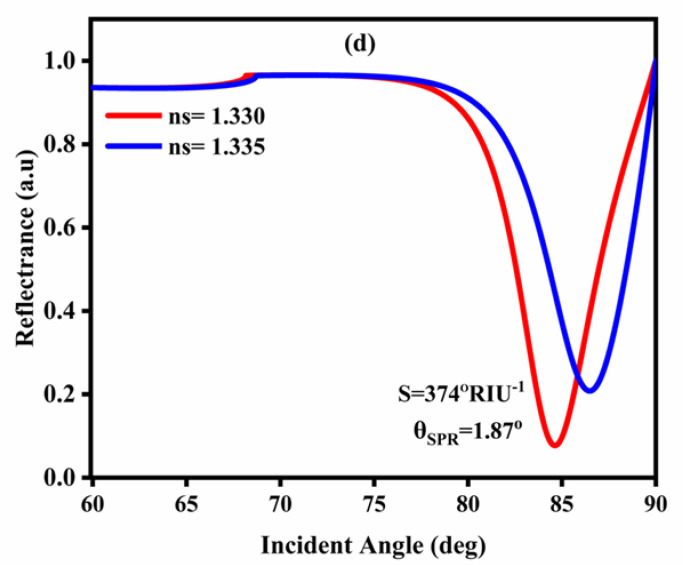
SPR reflectance curves: (**a**) design 1; (**b**) design 2; (**c**) design 3; (**d**) design 4.

The conventional and three revised characteristics plots of the specific SPR sensor are shown in Figure 2. For the conventional SPR sensor shown in Figure 2a, the variation of the refractive index is Δn = 0.005, while the Δθ and sensitivity and full width at half maximum (FWHM) are 0.72(deg), 144°/RIU, and 1.04677, respectively. Further, Figure 2b–d presents a comparison with the SPR reflectance curve from Figure 2a. The reflectance curves of SPR designs 2 (Ag/Al_2_O_3_) and 3 (Ag/Al_2_O_3_/Ni) are displayed in Figure 2b,c, respectively. The angular shifts (Δθ) for Figure 2b,c are 1.48 and 1.6, respectively, leading to sensitivities and FWHMs of 296° RIU^−1^ and 320° RIU^−1^, and 2.05852 deg and 2.25936 deg, respectively. Figure 2d suggests a superior resonance angle, sensitivity, and FWHM in contrast to the other 3 designs. Figure 2d has the most extreme sensitivity of 397°/RIU among all other structures. Table 3 shows not only the maximum sensitivity but also the maximum resonance angle and a larger figure of merit compared to the other 3 designs. 

Consequently, design 4 is considered the most reasonable decision among all designs.

## 3. Results and Discussion

The sensitivity properties of the biosensor with the changed Kretschmann design, which incorporates Al_2_O_3_ and Ni, are discussed here. To show how the sensitivity has reached the next level, from the reflectance curve the sensitivity is assessed according to the change in the resonance point. The excitement of the SPR causes a sharp drop in reflectance at a given point, which is clearly apparent. This event shows that the light is consumed by initiating the SPR in the biosensor arrangement, while the atom connection causes minor shifting in the refractive index of the sensor, which has a slight resonance dip around 1.87°. Thus, the design’s sensitivity is achieved (S_n_ = 374°/RIU) by utilizing the connected computation articulation S_n_ = S = $\frac{\Delta \theta_{\mathrm{res}}}{\Delta n}$as displayed in Figure 2.

The effects of the thickness variation of Al_2_O_3_ and Ni on the performance of the projected SPR sensor are shown in Table 4. The execution constraints of the proposed SPR sensor for the variety of thicknesses of Al_2_O_3_ (14–20 nm) and Ni (1, 3, 5 nm) are displayed in Table 4. The greatest sensitivity of 374°/RIU is obtained with a thickness for Al_2_O_3_ of 20 nm and for Ni of 1 nm at a working wavelength of 633 nm.

### 3.1. Use of CaF_2_ Crystal

The refractive index directly affects the performance of the SPR sensor. Since the shift with the incident point in the reflectance bend is high and a sharp plunge is acquired, a CaF_2_ crystal is utilized in the present situation. The sensitivity of this crystal material is incredible, with a lower RI than the CaF_2_ crystal. Therefore, the CaF_2_ glass crystal is at last utilized in the SPR sensor in the present theoretical investigation [36].

### 3.2. Performance Constraints of the SPR Sensor

The sensitivity, full width at half maximum, quality factor, detection accuracy, and limit of detection of the SPR sensor rely on certain variables. All of these constraints are dependent upon one another, and the reflectivity curve versus the incident angle determine the mathematical study of the SPR design.

### 3.3. Sensitivity (S)

The variation in resonance angle (Δθ_res_) with respect to the variation in the refractive index (Δn) decides the sensitivity and is defined as [37]:

(10) $$S=\frac{\Delta \theta_{\mathrm{res}}}{\Delta n}\;(\mathrm{Unit}:{}^{\circ}{\mathrm{RIU}}^{-1})$$

**Figure 3:**
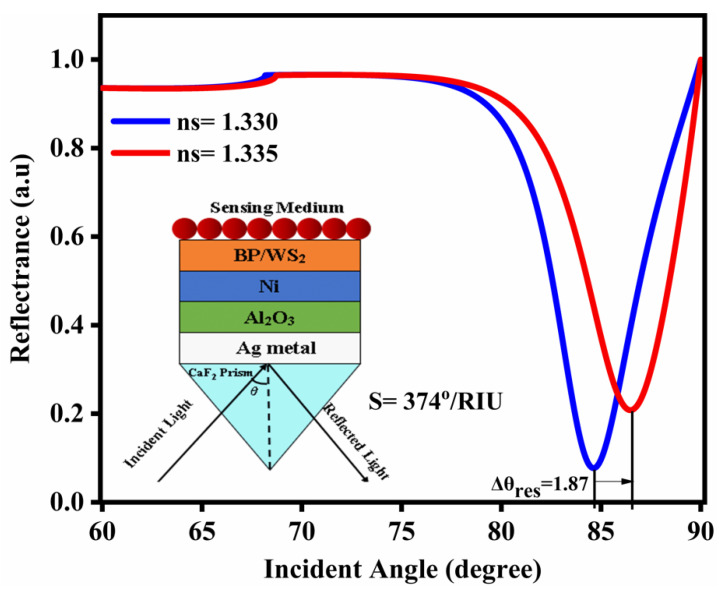
Variation of resonance angle vs. incident angle.

The variation of the reflectance with the incident angle for the proposed SPR sensor is shown in Figure 3.

Figure 3 shows the shifts in resonance angle with the incident angle at different RI values (n_SM_ = 1.330–1.335). From Figure 3, the greatest alteration in resonance angle (1.87) is acquired for the present SPR design. The most extreme change in the resonance point shows the adjustment of the coupling state of the surface plasmon wave (SPW). The device’s sensitivity should always be high. This means that the higher sensitivity sensor detects the minute variations in analyte (biomolecules) concentration, which shows that the sensor has superior sensing capabilities because it can easily detect minute RI variations in the structure.

### 3.4. Quality Factor (QF)

The division of sensitivity with the full width at half maximum is defined as the quality factor. It is also called the figure of merit (FOM). On the other hand, the quality factor is the multiplication of the sensitivity by the detection accuracy. The figure of merit (FOM) is a quantity used to characterize the performance as the device increases [38].

(11) $$\mathrm{QF}=\frac{\Delta \theta_{\mathrm{res}}}{\Delta n\;\times \mathrm{FWHM}}\;\;(\mathrm{unit}:\;{\mathrm{RIU}}^{-1})$$

**Figure 4:**
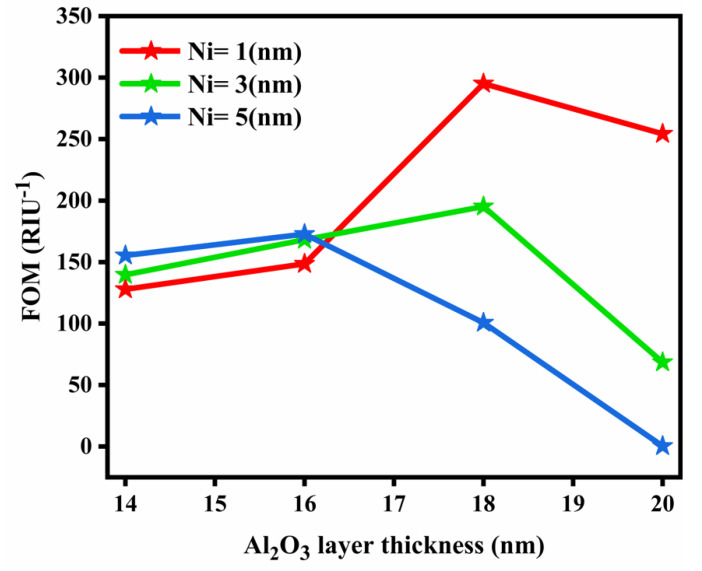
Variation of FOM vs. different layer thicknesses of Al_2_O_3_.

The variation of the FOM with the different layer thicknesses of Al_2_O_3_ is shown in Figure 4. It can be observed that the FOM is at its maximum for the Ni thickness of 1 nm.

### 3.5. Full Width at Half Maximum (FWHM)

The incidence angle changes at the halfway point of the reflectance curve can be used to calculate the full width at half maximum (FWHM), and its values should be low to boost the FOM. The FWHM assumes a significant role in the sensor’s execution because the majority of the parameters rely upon it. The width of the reflectance curve as the resonance angle shifts is measured by the FWHM. A low FWHM reduces the uncertainty in determining the resonance dip, and as a result improves the sensor’s resolution. It is defined by [39]:

(12) $$\mathrm{FWHM}=\frac{1}{2}\;(\theta_{\max}+\theta_{\min}),\;(\mathrm{unit}:\;\mathrm{degree})$$

**Figure 5:**
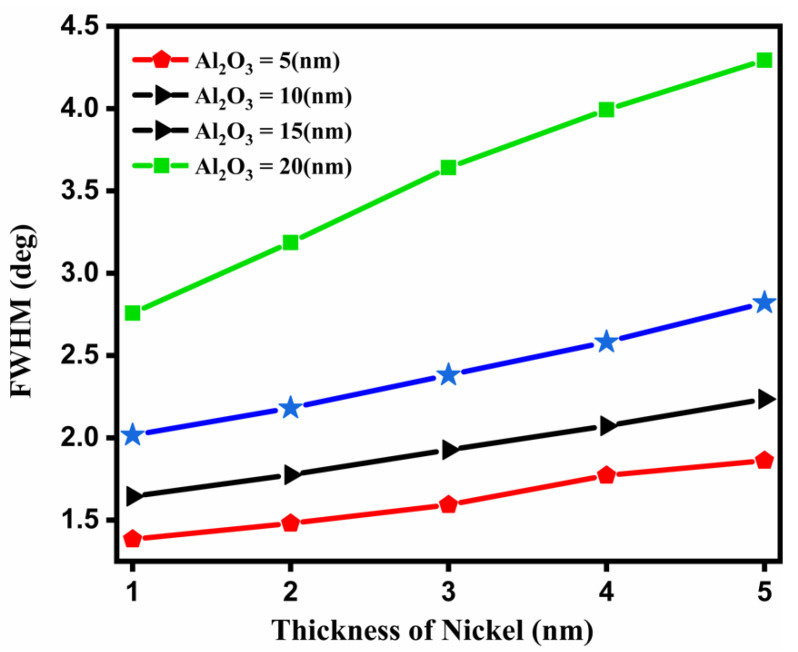
Variation of thickness of Ni vs. FWHM.

The FWHM with respect to the Ni thickness is shown in Figure 5.

Figure 5 shows that the variations of Ni vs. FWHM give the best result for the FWHM at the 20 nm thickness of Al_2_O_3_ when the thickness of Ni varies from 1 nm to 5 nm. The FWHM is also used to calculate the quality factor and detection accuracy.

### 3.6. Detection Accuracy (DA)

This is conversely connected with the full width at half maximum. It is also termed the signal-to-noise ratio, and the ratio of the signal to the noise should be high as possible to better the device quality. Basically, it shows how the noise level is impacting the device structure. It is defined as [30]:

(13) $$\mathrm{DA}=[\;\frac{1}{\;\mathrm{FWHM}}\;],\;({\mathrm{degree}}^{-1})$$

**Figure 6:**
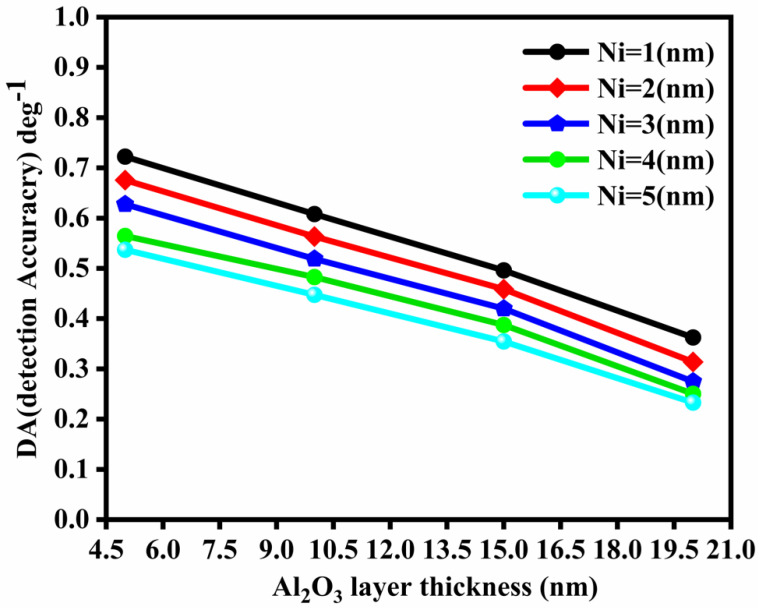
Plot of DA vs. different layer thickness for Al_2_O_3_.

The detection accuracy vs. layer thickness plot for Al_2_O_3_ is shown in Figure 6. It can be observed that the detection accuracy decreases with the layer thickness.

### 3.7. Limit of Detection (LOD)

This is the difference in biomolecule fixation or analyte concentration in the detecting area, and it is also defined as the proportion of progress in the RI (Δn) with the change in resonance angle (Δθres) [30]:

(14) $$\mathrm{LOD}=\frac{\Delta n}{{\Delta \theta }_{\mathrm{res}}}\times 0.001^{0}$$

where 0.001^0^ is a very small shift in the detecting medium.

### 3.8. The Impact of the Refractive Index on the Reflectance Curves of Different SPR Sensor Structures

**Figure 7:**
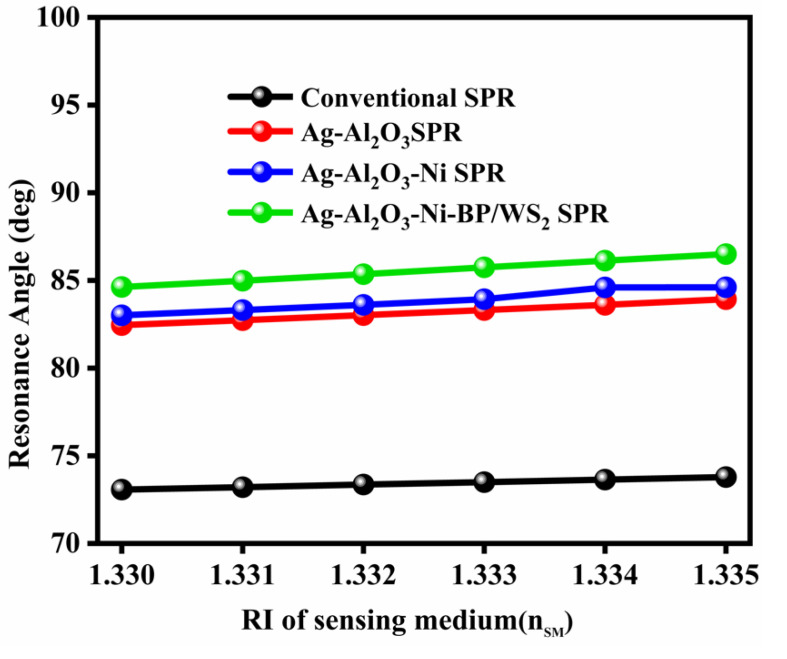
Variation of the resonance angle with Ri for different sensor designs.

The curves of SPR reflectivity for the sensor in the detecting medium with the RI range from 1.330 to 1.335. The proposed SPR curve for the changing RI of an analyte (n_s_ = 0.005) with adjustment of the resonance angle (1.87°), sensitivity (374°/RIU), quality factor (56.211 RIU^−1^), and DA (0.281 deg^−1^) for the recommended design might be found in the SPR reflectance bends. The sensitivity and resonance angle shift are significantly bigger than with conventional sensors, as displayed in Table 3. The variation of the resonance angle with the RI of the sensing medium for the conventional and proposed sensor designs are plotted in Figure 7.

### 3.9. Optimization of the Thicknesses of Al_2_O_3_ and Metal (Ni)

**Figure 8:**
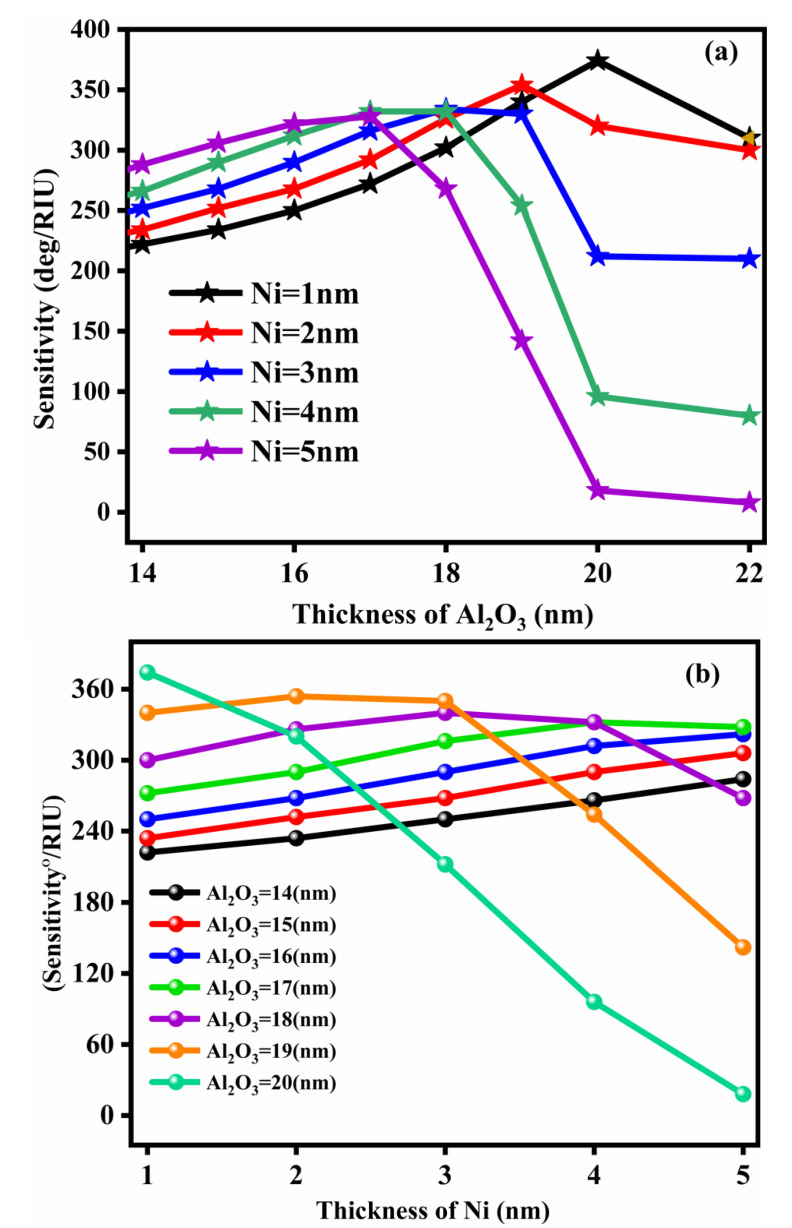
(**a**) Variation of sensitivity vs. Al_2_O_3_ (nm). (**b**) Variation of sensitivity vs. Ni (nm).

The refractive index of Al_2_O_3_ is small, so it should be selected because it minimizes the losses in performance of the biosensor, which is another characteristic contributing to the sensor’s improvement [32]. Since the Al_2_O_3_ layer has small damping properties with a higher penetration rate of the surface plasmon in the sensing medium, this property helps to stop the corrosion phenomena and enhances the performance of the SPR sensor [40]. On the other hand, the cost of the SPR sensor is reduced by using the ferromagnetic material nickel (Ni) because it has incredible magneto-optical characteristics, magnetic qualities, and small optical losses. At the optimum thickness of Ni (1 nm), the molecular absorption of light increases with minimum reflectance. The Ni layer is also used as a protective layer, which helps to raise the sensitivity of the SPR sensor [41]. Figure 8 shows a graph demonstrating the fluctuations in Al_2_O_3_ and Ni thickness and the varying sensitivities at different thicknesses. Figure 8a shows that the maximum sensitivity is achieved with a layer of Al_2_O_3_ of 20 nm. For metal, the best sensitivity is 374°/RIU (Ag). To determine the best sensitivity for the suggested SPR sensor, the thickness of Ni is optimized, as Figure 8b illustrates, whereby the sensitivity changes with the changes in Ni thickness. The optimal thickness ranges from 1 to 5 nm, with a maximum sensitivity of 374°/RIU and minimal reflection (R_min_). Consequently, the proposed SPR sensor shows the best results when the thickness of the Ni layer is 1 nm.

### 3.10. Parameter Analysis of the Projected SPR Biosensor

The design (CaF_2_/Ag/Al_2_O_3_/Ni/BlueP/WS_2_/sensing medium) arrangement has the most elevated sensitivity (374°RIU^−1^) for sensor applications. It is additionally advantageous for energizing surface plasmons by changing over from the crystal-directed mode to the surface plasmon polariton (SPP) mode proficiently. The performance parameters, sensitivity, and QF show increments with high RI values. However, the DA (detection accuracy) diminishes, which causes the increase in FWHM increments with a huge change in the reflectivity bend. The FWHM and DA have an opposite relationship.

### 3.11. Clarification of Transvers Magnetic (TM) Field and Penetration Depth

**Figure 9:**
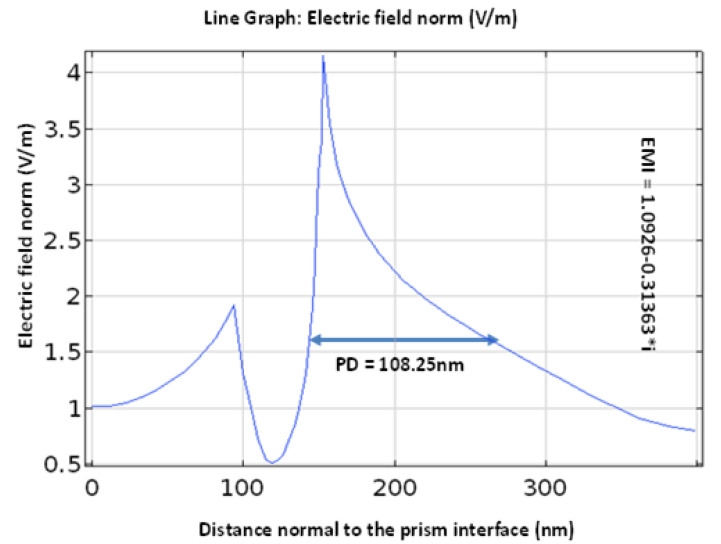
Penetration depth variation and transverse magnetic field.

The transverse magnetic (TM) field plot is discussed in this part. Utilizing the COMSOL Multiphysics programming software, the recommended SPR biosensor’s transverse magnetic (TM) field variation is shown in Figure 9. The TM field likewise helps with the estimation of a vital measurement, the sensor’s penetration depth. The distance of the electric field force diminishes to 1/e, which is called the penetration depth, and its value is 108.25 nm. Subsequently, when compared with past SPR sensor calculations, the proposed sensor has the best penetration depth, and along these lines is more delicate. To show the electromagnetic field, the effective mode index (EMI = 1.0926 − 0.31363 × i) is utilized [42].

## 4. Conclusions

In the work, the sensitivity was improved by utilizing a layer of Al_2_O_3_ over the metals silver (Ag) and nickel (Ni) on the top. A layer of 2D material, BlueP/WS_2_, was utilized to upgrade the sensitivity and safeguard the device from corrosion. The greatest sensitivity was found for Ag metal (374°/RIU) and the primary arrangement for the most extreme sensitivity was CaF_2_/Ag/Al_2_O_3_/Ni/BlueP/WS_2_/SM. The performance qualities of the heterostructure-based SPR sensors, such as FWHM, detection accuracy (DA), and LOD, showed great correlations with traditional sensors for the appropriate scope of the RI from 1.330 to 1.335. From the above study, the projected SPR sensor configuration has incredible sensitivity and could be utilized in the biosensing field.

## Figures and Tables

**Table 1:** Details of each layer of the proposed biosensor at 633 nm wavelength.

	Materials Used	Thickness (nm)	Refractive Index	References
1	CaF_2_ prism	100	1.4329	[28]
2	Ag metal	50	0.0803 + 1i × 4.234	[29]
3	Al_2_O_3_	20	1.7659	[30]
4	Nickel	1	0.031957 + 1i × 2.693	[31]
5	BlueP/WS_2_	0.75	2.48 + 1i × 0.170	[32]
6	Sensing medium	300	1.330 to 1.335	This work

**Table 2:** Comparative study of the proposed SPR model.

Device Structure	Assembling of Films
design 1 (Conventional SPR)	CaF_2_ crystal/Ag film/SM
design 2	CaF_2_ crystal/Ag film/Al_2_O_3_/SM
design 3	CaF_2_ crystal/Ag film/Al_2_O_3_/Ni/SM
design 4 (Proposed SPR)	CaF_2_ crystal/Ag film/Al_2_O_3_/Ni/BlueP/WS_2_/SM

**Table 3:** Comparative study of different proposed SPR sensors at a wavelength of 633 nm and Δn = 0.005.

Device Structure	Δθ (deg)	Sensitivity (° RIU^−1^)	FWHM (deg)	DA (deg^−1^)
Structure 1	0.72	144	1.04677	0.95531
Structure 2	1.48	296	2.05852	0.48578
Structure 3	1.6	320	2.25936	0.44260
Structure 4 (Proposed work)	1.87	374	2.75132	0.36346

**Table 4:** The optimized thickness values of Al_2_O_3_ and Ni with respect to the other parameters such as Δθ_SPR_, S, DA, FOM, and FWHM.

d(Al_2_O_3_) (nm)	d(Ni) (nm)	Δθ (deg)	S (°RIU^−1^)	DA (deg^−1^)	FWHM (deg)	FOM (RIU^−1^)
14	1	1.11	222	0.5190	1.9265	127.910
16	1	1.25	250	0.4753	2.1035	148.560
18	1	1.51	302	0.6471	1.5451	295.122
20	1	1.87	374	0.3635	2.7502	254.293
14	3	1.26	252	0.4399	2.2728	139.698
16	3	1.45	290	0.4000	2.2499	168.219
18	3	1.67	334	0.3498	2.8582	195.150
20	3	1.06	212	0.3048	3.2808	68.4950
14	5	1.44	288	0.3746	2.6690	155.381
16	5	1.61	322	0.3333	2.9995	172.834
18	5	1.34	268	0.2803	3.5672	100.670
20	5	0.09	18	0.2376	4.2070	0.38506

## Data Availability

Not applicable.
